# Epiglottic Abscess in an Adult Male: A Case Report

**DOI:** 10.7759/cureus.109004

**Published:** 2026-05-17

**Authors:** Wayne Eta

**Affiliations:** 1 Medicine, Université Libre de Bruxelles, Brussels, BEL

**Keywords:** adult epiglottitis, airway management, airway obstruction, deep neck infection, epiglottic abscess, epiglottitis, tracheotomy

## Abstract

Acute epiglottitis in adults is an uncommon but potentially life-threatening condition that can rapidly progress to airway compromise. Epiglottic abscess represents a rare and severe complication associated with increased morbidity and diagnostic challenges. We report a case of a 36-year-old male admitted to the emergency department in Belgium with progressive odynophagia, dysphonia, and hypersalivation despite prior oral antibiotic therapy. Laboratory investigations revealed a marked inflammatory response, including leukocytosis and elevated C-reactive protein. Contrast-enhanced computed tomography (CT) of the neck demonstrated a 30 mm epiglottic abscess extending toward the right tonsillar region, with associated mass effect on the supraglottic airway.

The patient was admitted to the intensive care unit for close airway monitoring and was managed conservatively with intravenous antibiotics, corticosteroids, and supportive care. Despite the size of the abscess, no surgical intervention was required. The patient showed rapid clinical improvement, with complete resolution of symptoms and no airway compromise. This case highlights the importance of early imaging and vigilant airway assessment in adult epiglottitis. It also supports the role of conservative management in carefully selected, clinically stable patients with epiglottic abscess.

## Introduction

Acute epiglottitis is a potentially life-threatening infection of the supraglottic structures that can lead to rapid airway obstruction if not promptly recognized and managed [1]. Although historically considered a pediatric disease, the epidemiology of *Haemophilus influenzae *type b has shifted following widespread immunization, with an increasing proportion of cases now occurring in adults [1].

In contrast to children, adult epiglottitis often presents with more subtle and insidious symptoms, including severe odynophagia, dysphagia, and dysphonia that may appear disproportionate to initial physical examination findings [2]. This variable presentation may delay diagnosis and complicate early management, particularly because the condition can mimic other causes of upper airway inflammation or deep neck infection. Delayed recognition increases the risk of complications, including abscess formation and acute airway compromise.

Epiglottic abscess formation is a rare but severe complication associated with deep neck space infection and significant morbidity. Early imaging, particularly contrast-enhanced computed tomography (CT), plays an important role in confirming the diagnosis, assessing the extent of disease, and distinguishing epiglottitis from other potentially life-threatening airway conditions [3]. Given the rarity of epiglottic abscess in adults, there remains limited consensus regarding optimal management strategies, particularly concerning the indications for surgical drainage versus conservative treatment. Careful airway assessment and close clinical monitoring are therefore essential. We report a case of a large epiglottic abscess in an adult patient successfully managed with conservative therapy alone.

## Case presentation

A 36-year-old male with no significant past medical history presented to the emergency department with a three-day history of worsening throat pain, dysphagia, and dysphonia. Symptoms persisted despite 24 h of oral amoxicillin/clavulanic acid therapy.

On admission, vital signs were stable (temperature: 36.6°C, heart rate: 78 beats per minute (bpm), blood pressure: 116/78 mmHg, oxygen saturation 99%) (Table 1). The patient exhibited odynophagia, hypersalivation, and a “hot potato” voice. He remained clinically stable in the sitting position but reported mild dyspnea when supine.

**Table 1:** Admission vital parameters. FC: cardiac frequency

Parameter	Value	Normal range
Heart rate (HR/FC) (beats per minute)	78	60-100
Blood pressure (mmHg)	116/78	~90/60-120/80
Temperature (°C)	36.6	36.0-37.5
Oxygen saturation (%)	99	≥95

Flexible nasofibroscopy revealed an inflamed and edematous epiglottis with posterior displacement and partial narrowing of the supraglottic airway (~40%). Vocal cord mobility was preserved. Laboratory investigations showed leukocytosis (14.18×10³/µL) with neutrophilia (74.6%) (Table 2). Hematological parameters demonstrated elevated white blood cell and neutrophil counts, with relative lymphopenia (Table 2). Coagulation studies revealed normal prothrombin time and INR, with elevated fibrinogen levels (Table 3). Biochemical analysis was largely unremarkable, except for mild hypokalemia and borderline low chloride levels (Table 4).

**Table 2:** Blood test results - hematology. MCV: mean corpuscular volume; MCH: mean corpuscular hemoglobin; MCHC: mean corpuscular hemoglobin concentration; abs: absolute

Test	Value	Normal range
White blood cells (×10³/µL)	14.18	4.0-11.0
Red blood cells (×10⁶/µL)	4.61	4.3-5.8
Hemoglobin (g/dL)	13.6	13.4-16.7
Hematocrit (%)	40.3	39.2-48.6
MCV (fL)	87.4	78.0-97.0
MCH (pg)	29.5	-
MCHC (g/dL)	33.7	-
Platelets (×10³/µL)	205	150-400
Neutrophils (%)	74.6	38.0-73.0
Eosinophils (%)	0.1	0.4-8.0
Basophils (%)	0.3	0.0-1.5
Lymphocytes (%)	15.3	20.0-50.0
Monocytes (%)	9.7	4.0-12.0
Neutrophils (abs) (×10³/µL)	10.59	1.4-7.7
Lymphocytes (abs) (×10³/µL)	2.17	1.0-4.8
Monocytes (abs) (×10³/µL)	1.37	0.2-1.0
Eosinophils (abs) (×10³/µL)	0.01	0.02-0.6
Basophils (abs) (×10³/µL)	0.04	0.0-0.11

**Table 3:** Coagulation profile demonstrating elevated fibrinogen levels with preserved PT and INR values. PT: prothrombin time; INR: international normalized ratio

Test	Value	Normal range
PT (%)	90	71.0-99.0
INR	1.1	0.8-1.2
Fibrinogen (g/L)	5.9	1.8-3.5

**Table 4:** Biochemical findings demonstrating preserved renal function with mild electrolyte abnormalities. eGFR: estimated glomerular filtration rate; MDRD: Modification of Diet in Renal Disease

Test	Value	Normal range
Hemolysis index	2.89	-
Icterus index	1.99	-
Lipemia index	4.96	-
Fasting glucose (mg/dL)	91	70-100
Sodium (mmol/L)	140	136-145
Potassium (mmol/L)	3.39	3.5-5.1
Chloride (mmol/L)	97	98-107
Bicarbonate (mmol/L)	28.8	22-29
Magnesium (mmol/L)	0.93	0.66-1.07
Osmolality (mOsm/kg)	299	281-303
Urea (mg/dL)	39.0	19.0-44.1
Creatinine (mg/dL)	0.94	0.72-1.25
Uric acid (mg/dL)	4.03	3.5-7.2
eGFR (MDRD) (mL/min/1.73 m²)	97	60.1-200.0

Liver function and inflammatory markers showed mildly elevated total and direct bilirubin, a markedly increased C-reactive protein level (205.9 mg/L), and mildly elevated creatine phosphokinase (Table 5). SARS-CoV-2 PCR testing was positive, while influenza and respiratory syncytial virus (RSV) tests were negative (Table 6).

**Table 5:** Liver enzyme and inflammatory marker abnormalities observed during clinical evaluation. CPK: creatine phosphokinase; ALT: alanine aminotransferase; GGT: gamma-glutamyl transferase; GPT: glutamate pyruvate transaminase; LDH: lactate dehydrogenase

Test	Value	Normal range
ALT (GPT) (U/L)	19	0-55
GGT (U/L)	25	0-55
Alkaline phosphatase (U/L)	97	50-116
LDH (U/L)	181	125-220
Total bilirubin (mg/dL)	1.66	0.2-1.2
Direct bilirubin (mg/dL)	0.57	0.0-0.5
Lipase (U/L)	22	0-64
CRP (mg/L)	205.9	0-5
CPK (U/L)	259	30-200

**Table 6:** PCR respiratory panel. RSV: respiratory syncytial virus; Ct: cycle threshold

Test	Result	Reference/normal
SARS-CoV-2 (PCR combo)	Detected	Not detected
SARS-CoV-2 Ct value	32.10	-
Influenza A (PCR combo)	Not detected	Not detected
Influenza A1 Ct	0.00	-
Influenza A2 Ct	0.00	-
Influenza B (PCR combo)	Not detected	Not detected
Influenza B Ct	0.00	-
Respiratory syncytial virus A+B (PCR combo)	Not detected	Not detected
RSV A+B Ct	0.00	-

**Figure 1:**
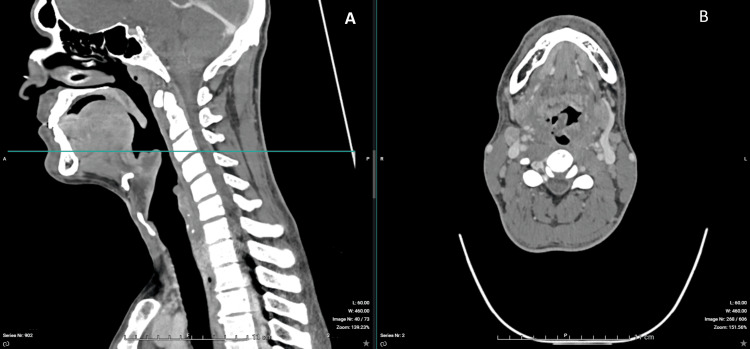
Contrast-enhanced CT imaging of the epiglottis. (A) Sagittal view (left) demonstrating a markedly thickened and edematous epiglottis causing significant mass effect on the airway. The horizontal line indicates the level of the axial section. (B) Axial view (right) demonstrating severe narrowing of the supraglottic lumen, consistent with impending airway compromise.

**Figure 2:**
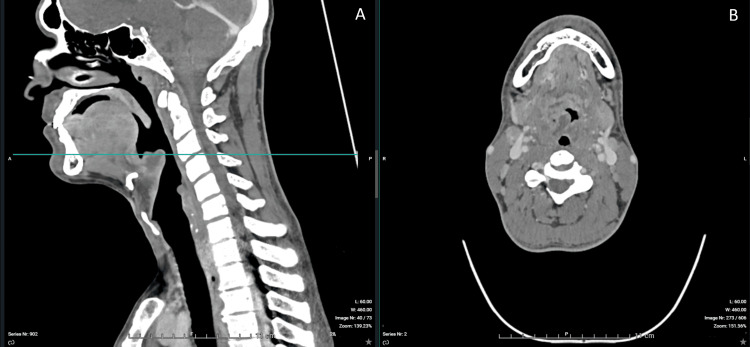
CT imaging of supraglottic airway narrowing. (A) Sagittal view (left) demonstrating irregular narrowing of the supraglottic airway due to inflammatory edema. The horizontal line indicates the level of the axial section. (B) Axial view (right) demonstrating distortion of the airway lumen, which remains partially patent.

**Figure 3:**
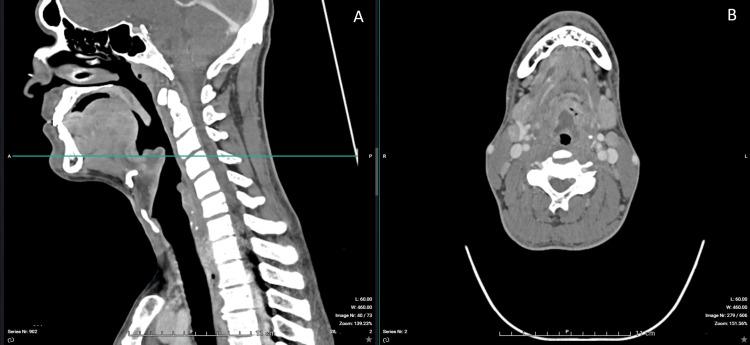
CT features of epiglottic abscess. (A) Sagittal view (left) demonstrating a hypodense collection within the epiglottis. The horizontal line indicates the level of the axial section. (B) Axial view (right) demonstrating internal air foci, suggestive of abscess formation with possible anaerobic infection.

**Figure 4:**
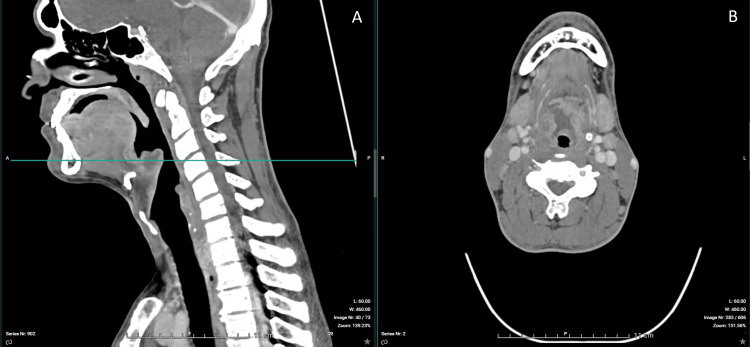
Extension of the inflammatory process. (A) Sagittal view (left) demonstrating extension of inflammation toward the right tonsillar region. The horizontal line indicates the level of the axial section. (B) Axial view (right) demonstrating surrounding soft tissue infiltration and asymmetry.

**Figure 5:**
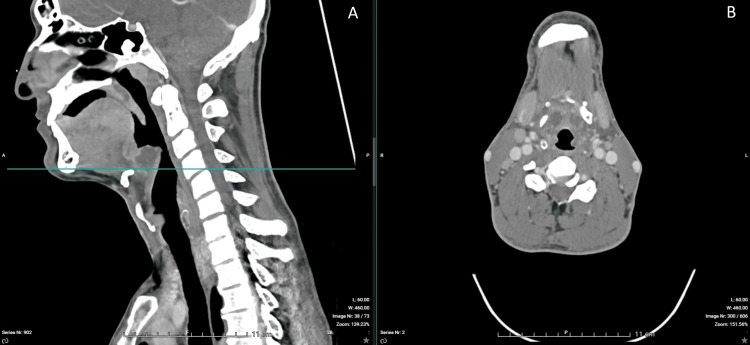
Extent of epiglottic enlargement. (A) Sagittal view (left) demonstrating significant epiglottic thickening. The horizontal line indicates the level of the axial section. (B) Axial view (right) demonstrating marked reduction of the supraglottic lumen due to mass effect.

**Figure 6:**
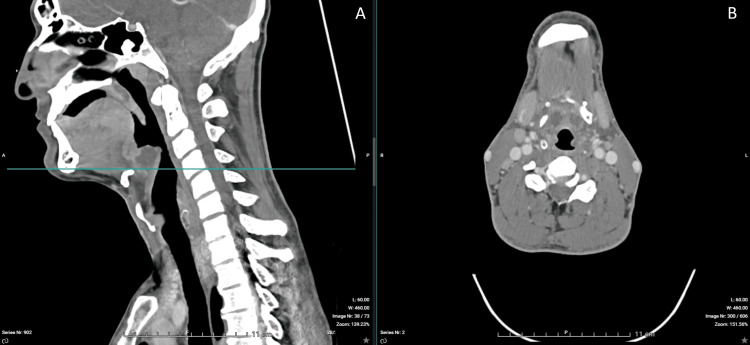
Epiglottic abscess with airway compromise. (A) Sagittal view (left) demonstrating a markedly thickened and edematous epiglottis with an intralesional hypodense collection. The horizontal line indicates the level of the axial section. (B) Axial view (right) demonstrating posterior displacement of the epiglottis and significant narrowing of the supraglottic airway.

Contrast-enhanced CT imaging confirmed a 30-mm epiglottic abscess with extension toward the right tonsillar region and associated cervical lymphadenopathy (Figures 1-6). The patient was admitted to the intensive care unit for close airway monitoring, with contingency planning for possible surgical airway intervention, including tracheotomy. He was treated with intravenous ceftriaxone, metronidazole, corticosteroids, and supportive care. No surgical drainage was performed due to clinical stability. The patient improved progressively, with complete resolution of symptoms and no airway compromise.

## Discussion

Adult epiglottitis is increasingly recognized in the post-*Haemophilus influenzae *type b (Hib) vaccination era, with incidence estimates ranging from one to four cases per 100,000 individuals annually [1]. Unlike pediatric cases, adult presentations are often less acute but may be complicated by abscess formation due to delayed diagnosis [2]. The microbiology of adult epiglottitis is typically polymicrobial, involving Streptococcus species, *Staphylococcus aureus*, and anaerobic organisms [3]. The presence of intralesional air in this case suggests anaerobic involvement.

Clinically, adult epiglottitis presents with severe odynophagia, dysphagia, and dysphonia, often disproportionate to physical examination findings, highlighting the importance of imaging in diagnosis [4]. The marked inflammatory response observed in this patient, including leukocytosis, neutrophilia, and markedly elevated C-reactive protein levels, was consistent with a severe infectious process and supported the need for close clinical and airway monitoring. Although these findings are nonspecific, they contributed to the overall assessment of disease severity and the decision to initiate broad-spectrum intravenous antibiotic therapy and intensive care surveillance.

Contrast-enhanced CT is the imaging modality of choice for identifying epiglottic abscess and assessing extension into deep neck spaces. In this case, CT excluded involvement of the danger space, which is associated with worse outcomes [5]. Airway management remains the primary concern. However, routine intubation is not always required in adults, as only a minority of patients require airway intervention [2]. Close monitoring in an intensive care setting is, therefore, appropriate for stable patients.

Empirical antibiotic therapy should provide coverage for both aerobic and anaerobic organisms. The combination of ceftriaxone and metronidazole used in this case is consistent with current recommendations [6]. The role of corticosteroids remains controversial, although they may contribute to reducing airway edema [4]. While epiglottic abscess has traditionally been managed surgically, recent evidence supports conservative management in selected cases with stable airways and favorable clinical response [6]. The coexistence of SARS-CoV-2 infection in this patient may have contributed to the inflammatory response and upper airway edema, although the exact relationship remains unclear [7].

## Conclusions

Epiglottic abscess in adults is a rare but potentially life-threatening condition requiring prompt recognition and multidisciplinary management. Contrast-enhanced CT imaging plays a key role in the diagnosis and assessment of disease extent. While airway protection remains the primary priority, conservative treatment with antibiotics and corticosteroids may be effective in selected, clinically stable patients.

Clinicians must remain vigilant for potential complications, including rapid airway obstruction, abscess rupture with aspiration, and extension into deep neck spaces. In cases of clinical deterioration, urgent airway intervention is required. Tracheotomy, although infrequently performed, remains a definitive and life-saving option when endotracheal intubation is not feasible. Careful patient selection, close monitoring, and readiness for escalation of airway management are essential to ensure favorable outcomes.
